# Changes in diet associated with cancer: An evolutionary perspective

**DOI:** 10.1111/eva.12465

**Published:** 2017-05-20

**Authors:** Frédéric Thomas, Sophie Rome, Frédéric Mery, Erika Dawson, Jacques Montagne, Peter A. Biro, Christa Beckmann, François Renaud, Robert Poulin, Michel Raymond, Beata Ujvari

**Affiliations:** ^1^ CREEC/MIVEGEC UMR IRD/CNRS/UM 5290 Montpellier Cedex 5 France; ^2^ CarMen (UMR INSERM 1060, INRA 1397, INSA) Faculté de Médecine Lyon‐Sud Université de Lyon Oullins France; ^3^ Evolution, Génomes, Comportement and Ecologie CNRS, IRD Université Paris‐Sud, Université Paris‐Saclay Gif‐sur‐Yvette France; ^4^ Institute for Integrative Biology of the Cell (I2BC) CNRS Université Paris‐Sud, CEA, UMR 9198 Gif‐sur‐Yvette France; ^5^ Centre for Integrative Ecology School of Life and Environmental Sciences Deakin University Waurn Ponds VIC Australia; ^6^ Department of Zoology University of Otago Dunedin New Zealand; ^7^ Institute of Evolutionary Sciences University of Montpellier Montpellier France

**Keywords:** malignant cells, manipulation, nutrition, self‐medication, resistance, tolerance

## Abstract

Changes in diet are frequently correlated with the occurrence and progression of malignant tumors (i.e., cancer) in both humans and other animals, but an integrated conceptual framework to interpret these changes still needs to be developed. Our aim is to provide a new perspective on dietary changes in tumor‐bearing individuals by adapting concepts from parasitology. Dietary changes may occur alongside tumor progression for several reasons: (i) as a pathological side effect with no adaptive value, (ii) as the result of self‐medication by the host to eradicate the tumor and/or to slow down its progression, (iii) as a result of host manipulation by the tumor that benefits its progression, and finally (iv) as a host tolerance strategy, to alleviate and repair damages caused by tumor progression. Surprisingly, this tolerance strategy can be beneficial for the host even if diet changes are beneficial to tumor progression, provided that cancer‐induced death occurs sufficiently late (i.e., when natural selection is weak). We argue that more data and a unifying evolutionary framework, especially during the early stages of tumorigenesis, are needed to understand the links between changes in diet and tumor progression. We argue that a focus on dietary changes accompanying tumor progression can offer novel preventive and therapeutic strategies against cancer.

## 1 INTRODUCTION

Understanding the roles of diet and nutrition in malignant tumor development and progression (i.e., cancer) is central for several reasons (Mayne, Playdon, & Rock, 2016). For instance, the American Institute for Cancer Research and the World Cancer Research Fund estimated that 30%–40% of all cancers could be prevented by adopting a suitable diet, together with physical activity and maintenance of appropriate body weight (i.e., below 22.5 kg/m^2^; Glade, 1999). Similarly, there is increasing evidence that some diets are more favorable than others to the likelihood of recovery for cancer patients (Donaldson, 2004). In addition to deliberate diet manipulations designed to alleviate cancer risk, it has been shown that cancer can induce changes in human food consumption and/or diet preference. For example, anorexia (reduced appetite) is a general symptom in patients with advanced cancer, which also significantly contributes to cancer cachexia, a wasting process that results in a dramatic loss of muscle and adipose tissue mass (Davis, Dreicer, Walsh, Lagman, & LeGrand, 2004; DeWys, 1979; Tisdale, 2001). Although less frequent, certain cancers, such as malignant gastric ghrelinoma, can conversely induce increased appetite (Tsolakis et al., 2004). The changes in diet preference that occur with cancer and the proximate reasons underlying them have been intensively studied (Box 1), but the possibility that dietary changes themselves serve a function has only recently been considered seriously (Tissot et al., 2016).

Box 1 **Why do changes in diet occur for cancer patients?**

1 Both cancer and cancer treatments can cause dietary changes through a variety of processes, affecting for instance taste, smell, appetite, satiety, and/or the ability to absorb the nutrients from food (von Meyenfeldt, 2005; Van Cutsem & Arends, 2005). For instance, patients with cancer may suffer from the presence of abnormal taste sensation (even before chemotherapy treatment) which includes increased desire for sweet foods (Elkort, Baker, Vitale, & Cordano, 1981), general aversion to sweet flavors (dysgeusia) (Nakazato, Imai, Abe, Tamura, & Shimazu, 2006), increased sensitivity to bitterness, and aversion to meat (DeWys & Walters, 1975). Interestingly, patients on chemotherapy are less likely to display a distinct preference for high‐ or low‐glucose diet compared to those not on chemotherapy (Trant, Serin, & Douglass, 1982). Tumors, depending on their size and location, may also partly block the digestive system (e.g., esophageal cancer, stomach cancer, or bowel cancer) or render swallowing difficult and painful (throat or mouth cancers). Some cancers may cause the spleen to become larger and exert pressure on the stomach, thereby creating a feeling of fullness. Other factors, like chronic pain, analgesic treatments, tiredness, or cancer‐associated depression, fear, and anxiety are known to result in a decreased appetite.

From an evolutionary perspective, an organism's feeding decisions result from various internal and external stimuli that are integrated into behavioral responses that allow individuals to navigate the spectrum of trade‐offs among competing demands for longevity, reproduction, and immune function (Wong et al., 2015). Modern biology also recognizes that the body of multicellular organisms is composed, in addition to host cells, of a diversity of living entities (e.g., microbes, parasites) competing for nutritional resources (Consortium 2012; McFall‐Ngai et al., 2013). Evolutionary conflict between the host and these living entities may lead to cravings and cognitive conflict with regard to eating behaviors (Alcock, Maley, & Aktipis, 2014). For instance, what a host eats alters the microbiome's composition and, as a consequence, also affects microbiome‐derived signals that in turn result in changes in immune and metabolic functions (Wong et al., 2015). These feedback signals from the microbiome can subsequently influence the host's eating behavior, along with direct processes by the microbes such as the provisioning of nutrients as well as competition for nutrients ingested by the host (Douglas, 2009, 2011; Kostic, Howitt, & Garrett, 2013; Nicholson et al., 2012; Wong et al., 2015). Changes in diet are also frequently reported in parasitized animals, being host adaptations against parasites or conversely, parasitic manipulations favoring transmission or host exploitation (Hughes, Brodeur, & Thomas, 2012; Poulin, 2007).

Despite important differences between parasites and malignant cells, a close comparison can be drawn between cancer and infectious diseases due to the similarities in their life history and their impact on the host's body. Malignant cells proliferate inside their host body and exploit them for resources. However, in spite of this dependence, cancer cells impair vigor and health of the host body. On this basis, it is expected that several of the phenotypic responses observed in the context of host–parasite interactions should also be relevant in the context of cancer (Ujvari, Beckmann, et al., 2016; Vittecoq et al., 2015). Therefore, we propose to apply the conceptual framework used in parasitology to understand cancer‐induced dietary changes. This framework suggests that dietary changes are (i) the nonadaptive result and/or by‐product of infection, (ii) the result of parasite (here tumor) manipulation, or (iii) indicative of host adaptation (Poulin, 1995).

As far as host adaptations are concerned, hosts employ a range of strategies to defend themselves against infectious agents, namely avoidance, resistance, and tolerance (Medzhitov, Schneider, & Soares, 2012; Råberg, Graham, & Read, 2009; Rausher, 2001). Briefly, avoidance behavior avoids contact with pathogens, whereas resistance minimizes the success of a parasite by preventing its establishment or inhibiting its growth (i.e., parasite burden). In contrast, a tolerant host, although susceptible, acts to minimize the fitness effects of infection, by repairing the damages incurred, but without directly affecting parasite fitness. Distinguishing between resistance and tolerance is not a semantic issue, but rather a necessity as their ecological and evolutionary consequences are different. Resistance imposes selection on the parasite leading to antagonistic coevolution between host and parasite. Conversely, tolerance by definition does not have any negative effect on the performance of the parasite, and so there should not be any selection on the parasite to overcome this type of defense (Råberg et al., 2009). To our knowledge, these concepts have not yet been applied in the context of understanding the interaction between host diet and resistance or tolerance to cancer.

### 1.1 When do changes in diet appear during tumorigenesis?

While the time elapsing from the appearance of the first neoplastic cells to the development of a metastatic cancer may vary from months to years, even decades, most dietary changes in tumor‐bearing individuals are only studied once a cancer is diagnosed from tumors large enough to be detected, and/or after treatments have started. As a result, we know very little about the occurrence and timing of diet changes during tumorigenesis. Similarly, current epidemiological investigations demonstrating relationships between dietary factors and cancer cannot conclusively determine whether changes in diet are a cause or a consequence of cancer progression (Mayne et al., 2016). There is thus an urgent need to extend the investigation to a broad range of species with inducible cancer models to enable us to acquire systematic information on changes in diet from the early onset of the tumorigenesis.

### 1.2 Possible determinants of changes in diet in cancer patients

#### 1.2.1 Pathological side effects

The most parsimonious hypothesis for phenotypic alterations like differences in diet between healthy and tumor‐bearing individuals needs only involve side effects of pathology that may or may not be coincidentally beneficial for the host or the tumor. Within the long list of proximate mechanisms responsible for changes in diet in tumor‐bearing individuals (Box 1), several are likely to correspond to such nonfunctional by‐products. For instance, reduced appetite resulting from tumors physically blocking the digestive system is likely to be an unavoidable consequence of malignant growth. Because hosts with malnutrition have reduced immunity, this can also indirectly favor malignant growth. In practice, there is probably no straightforward experimental way of distinguishing between an advantageous by‐product and an advantageous direct product of oncogenic selection (i.e., evolutionary selection on cells during oncogenesis).

#### 1.2.2 Tumor manipulation

Parasite‐induced alterations of host phenotype have been reported in a wide range of protozoan and metazoan parasites (Hughes et al., 2012; Moore, 2002). Tissot et al. (2016) recently explored this phenomenon in the context of malignancies, arguing that oncogenic selection, even though it occurs on a maximum of a few decades for the majority of cancers (Arnal et al., 2015; Ujvari, Gatenby, & Thomas, 2016), could favor variants that are able to manipulate their host similar to “true” parasitic/symbiotic organisms. Concerning eating behaviors, cancer cells could, for instance, modify host appetite in a quantitative/qualitative way that is favorable for the tumor, by inducing cravings for foods that give malignant cells fitness advantages over healthy cells, and/or inducing dysphoria, a general feeling of unease, until the hosts prioritize foods that enhance their fitness (Tissot et al., 2016). Manipulating host preference for novel food would be expected notably if malignant cells need nutrients that are not routinely consumed by the host. Although cancer cells have a specific metabolism (Phan, Yeung, & Lee, 2014), they were, however, derived from healthy cells and are therefore likely to broadly rely on the same resources. A more plausible outcome is therefore a scenario of indirect manipulation in which malignant cells overexploit the resources that are present, which may result in alteration of host homeostasis (Box 2), and hence favor the evolution of compensatory responses. For instance, as a compensatory response, tumor‐bearing individuals might have higher preference for sugar or other high‐energy substances, a portion of which is subsequently hijacked by the tumor. For example, cancer cells are known not only to grow in the vicinity of adipose tissue, but also to actively exchange metabolites with cancer‐associated adipocytes (CAA) (Nieman, Romero, Van Houten, & Lengyel, 2013). CAA create a resource‐rich microenvironment for cancer cells that require high amounts of fatty acids and cholesterol to sustain their elevated growth rate. Given that adipocytes are central in lipoprotein traffic and that adipose tissue depots are abundant in obese individuals (Louie, Roberts, & Nomura, 2013), cancer may potentially drive dietary choices, satiety, and hence excess weight gain.

Box 2 **Cachexia is an example of tumor host manipulation which leads to complex metabolic disorder that involves loss of adipose and skeletal muscle mass to produce energy and substrate for tumor growth**

1 TAG: Triacylglycerol; PIF: Proteolysis‐inducing factor; LMF: Lipid‐mobilizing factor; TNF‐a: Tumor necrosis factor alpha; FFA: free fatty acids (see, for instance, Tisdale, 2001).

**Figure 1:**
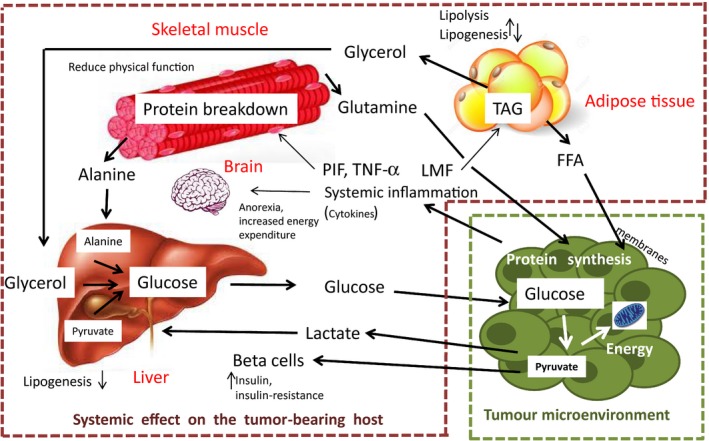
Systemic effect on the tumor‐bearing host

From a mechanistic point of view, to manipulate eating habits malignant cells could potentially produce toxins that alter mood, influence reward and satiety pathways, change receptors (including taste receptors), and hijack the vagus nerve, i.e., the neural axis between the gut and the brain (Alcock et al., 2014). Although undoubtedly more empirical evidence would be necessary, at least one study supports the manipulation hypothesis. For instance, Huang et al. (2016) found that tumor‐induced hyperlipidemia encompasses a feed‐forward loop that reprograms hepatic lipoprotein homeostasis in part by providing LDL cholesterol that supports tumor growth (i.e., tumor‐induced hyperlipidemia contributes to tumor growth during cachexia).

#### 1.2.3 Host adaptation against cancer progression

##### Resistance

Change in diet as a self‐medication strategy against cancer is plausible for at least two reasons. First, this defensive strategy is widespread in the animal kingdom against infectious agents (Clayton & Wolfe, 1993; Huffman, 2001; Lozano, Milinski, Slater, & Møller, 1998; Raubenheimer & Simpson, 2009; de Roode, Lefèvre, & Hunter, 2013; Singer, Mace, & Bernays, 2009). As ecosystems contain a plethora of food types (e.g., leaves, bark, fungi) with potential effective cancer treatment properties, it is theoretically possible that self‐medication via changes in diet could have evolved to control and/or to suppress malignancies associated with fitness reductions (Vittecoq et al., 2015). For instance, antioxidants including certain carotenoids play a key role in mopping up free radicals that otherwise damage DNA sequences (Møller et al., 2000), and animals show a strong preference for food containing antioxidants (Senar et al., 2010). In addition, as cancer progression is also influenced by diet parameters (Mayne et al., 2016), tumor‐bearing individuals could potentially have (at least at certain periods of the carcinogenesis) some control over tumor progression through a change in diet. Weight loss could represent, at least at the beginning of the disease, a form of host adaptation to prevent tumor development (resistance) (Grosvenor, Bulcavage, & Chlebowski, 1989). Indeed, glucide restriction associated with weight loss is known to decrease insulin levels and the progression of several different types of cancer, with only a minor effect on homeostasis, because during starvation glucose utilization is replaced by ketones derived from fat. This leads to restriction of gluconeogenesis from amino acids by the liver and maintenance of muscle mass in contrast to what happens during cachexia (Box 2).

A substantial portion of human cancer results from parasites (defined broadly to include multicellular, cellular, and subcellular organisms) (see Ewald, 2009; Ewald & Swain Ewald, 2017). Therefore, dietary associations with parasites may not only be models for dietary associations with cancer, but also a cause; for example, anorexia might help control cancer by controlling the parasites that are causing the cancer.

##### Tolerance

Theory predicts that hosts unable to resist cancer progression by other means (e.g., immunological resistance) will be favored by selection if they partly compensate the fitness losses due to cancer. At least two mechanisms are possible for this strategy: (i) investing more resources into immediate reproduction to maximize fitness before dying (e.g., adjustments of life history traits (Arnal et al., 2017; Ujvari, Beckmann, et al., 2016)) and (ii) repairing/compensating the damages caused by tumor progression to allow reproduction for as long as possible. These two processes, because they act to minimize the fitness effects of cancer without directly impairing tumor progression, could be considered as forms of tolerance to cancer. Both responses are likely to rely on change in diet because they necessitate novel energetic requirements (see, for instance, in the context of parasites: Lee, Cory, Wilson, Raubenheimer, & Simpson, 2006; Ponton et al., 2011; Mason, Smilanich, & Singer, 2014; Povey, Cotter, Simpson, & Wilson, 2014.

Because several of the basic requirements of neoplastic cells are likely to be the same as those of healthy cells, changes in diet that could be beneficial for the host to tolerate a cancer could also be beneficial for malignant progression. However, provided the net fitness benefits for tumor‐bearing individuals are high enough, tolerance can remain the best option from an evolutionary perspective even if the host's death is anticipated. This is because tolerance, by keeping the organisms capable of reproduction, would give a significant advantage when the selection is intense (first half of the reproductive period) and a disadvantage mainly when the selection is weak (second half or end of the reproductive period). From the cancers’ point of view, this scenario mimics a manipulative process from the tumor (i.e., the change in diet results in a higher tumor growth), but it is primarily a host adaptation to cancer to maximize fitness, by making the best of a bad situation. When host compensatory responses mitigate the costs of malignant proliferation without impairing tumor progression, natural selection acting on the host and oncogenic selection acting on cells during oncogenesis should favor genes involved in this interaction.

## 2 IMPLICATIONS FOR PREVENTIVE AND THERAPEUTIC STRATEGIES

Knowing why, when, and how cancer cells induce changes in diet could be very valuable in combating cancer. Currently, changes in diet are mainly studied once tumors are detected, which is usually late in the tumorigenesis process. A better understanding of the change in diet occurring before tumors are detectable could potentially improve our understanding of some subtle interactions occurring between malignant cells and their host during the first steps of the tumorigenesis. Such knowledge would also potentially permit the identification of tumor‐bearing subjects, particularly in the case of cancers with long latencies. Early changes in diet and modifications of metabolic parameters (blood glucose or lipids levels) may indeed indicate that the tumor is hijacking the host's metabolism for its own energy demand. For instance, experimental models show that modification of insulin levels associated with glycemic dysregulation appears early during cancer development: Pancreatic carcinogenesis (Li, 2012) and breast cancer are preceded by modifications of blood insulin levels (Ferroni et al., 2016). Insulin increases glucose use and may also promote cell proliferation, both of which are important for tumor development and progression. In the case of breast cancers, insulin levels are associated with disease stage (Ferroni et al., 2016) and have recently been shown to provide prognostic information to improve breast cancer detection and management. Apart from biochemical parameters, modifications of energy balance and thus body weight might also indicate early nondetectable tumors. For example, pancreatic cancers and type I diabetes are frequently associated with weight loss that is different from cachexia and predicts the development of pancreatic cancer in these diabetic patients (<3 years) (Wang, Herrington, Larsson, & Permert, 2003). Another promising direction in an applied perspective concerns the links between diet, cancer, and body odor. Indeed, while cancer patients frequently change odor from very early during tumor progression (e.g., Hackner & Pleil, 2017; Horvath, Järverud, Järverud, & Horváth, 2008; Jezierski, Walczak, Ligor, Rudnicka, & Buszewski, 2015; Pickel, Manucy, Walker, Hall, & Walker, 2004), these alterations could be closely linked to changes in dietary preference (e.g., Beauchamp, 1976; Ferkin, Sorokin, Johnston, & Lee, 1997; Havlicek & Lenochova, 2006).

Even if it is clear that cancer is associated with metabolic reprogramming and change in diet, it is actually not clear whether these changes only result from cancer progression or represent a form of adaptation of the host to eradicate malignant progression and/or tolerate it.

Accordingly, determining whether changes in diet in cancer individuals benefit the host, cancer cells, both (e.g., tolerance), or neither may help inform cancer therapy (see also Ewald, 1980). This knowledge together with the developments of tools for monitoring specific change in diet should permit the identification of actual life periods when the risk of invasive cancer initiation is the highest. It would also be informative to determine whether oncogenic progression, from precancerous lesions to metastasis, relies on a more or less constant/obligatory sequence of change in diet, which could potentially be altered by adapted therapies. There is currently a pressing need to understand the selective pressures and proximate factors shaping the link between tumor progression, local manipulative processes by malignant cells (i.e., angiogenesis), and diet changes in tumor‐bearing individuals. Depending on these links, the next step will be to integrate change in diet as a factor in the design of preventive strategies and/or cures.

## 3 CONCLUDING REMARKS

While changes in diet are well known in tumor‐bearing individuals, a conceptual framework is needed to interpret them. In addition to addressing this gap in knowledge, we highlight here that natural selection could theoretically favor changes in diet that alleviate the fitness costs of cancer but that favor at the same time its long‐term progression. This is not paradoxical from an evolutionary perspective given that organisms are the result of natural selection to secure the propagation of genes, but not for maintaining good health as long as possible. However, conversely to resistance, such tolerance has dramatic consequences for human health after the reproductive period. Elucidating the relative importance of these two types of defenses is therefore key from an applied medical perspective.

Recently, Wong et al. (2015) provided an integrative framework to study the interactions between the nutrient environment, the metabolic and behavioral responses of the host, and the microbiome. Their approach, called nutritional geometry, integrates and maps multiple aspects of the host and microbial response in multidimensional nutrient intake spaces. While we fully support this view, we also believe that malignant cells should be considered as full players in these interactions. Understanding changes in diet during tumorigenesis within a unifying evolutionary framework is still in its infancy. Such knowledge permits not only to highlight some of the fundamental processes governing cancer dynamics, but also to identify tools that are insufficient or missing in evolution's toolbox to eradicate it.
